# Online Biomass Monitoring Enables Characterization of the Growth Pattern of *Aspergillus fumigatus* in Liquid Shake Conditions

**DOI:** 10.3390/jof8101013

**Published:** 2022-09-27

**Authors:** Ingo Bauer, Beate Abt, Annie Yap, Bernd Leuchtle, Hubertus Haas

**Affiliations:** 1 Institute of Molecular Biology, Biocenter, Medical University of Innsbruck, Innrain 80-82, 6020 Innsbruck, Austria; 2 SBI, Scientific Bioprocessing, 520 William Pitt Way, Pittsburgh, PA 15238, USA

**Keywords:** fungi, molds, *Aspergillus fumigatus*, liquid shake culture, flask culture, online monitoring, biomass monitoring, backscatter, bioprocess automation

## Abstract

Numerous filamentous fungal species are extensively studied due to their role as model organisms, workhorses in biotechnology, or as pathogens for plants, animals, and humans. Growth studies are mainly carried out on solid media. However, studies concerning gene expression, biochemistry, or metabolism are carried out usually in liquid shake conditions, which do not correspond to the growth pattern on solid media. The reason for this practice is the problem of on-line growth monitoring of filamentous fungal species, which usually form pellets in liquid shake cultures. Here, we compared the time-consuming and tedious process of dry-weight determination of the mold *Aspergillus fumigatus* with online monitoring of biomass in liquid shake culture by the parallelizable CGQ (“cell growth quantifier”), which implements dynamic biomass determination by backscattered light measurement. The results revealed a strong correlation of CGQ-mediated growth monitoring and classical biomass measurement of *A. fumigatus* grown over a time course. Moreover, CGQ-mediated growth monitoring displayed the difference in growth of *A. fumigatus* in response to the limitation of iron or nitrogen as well as the growth defects of previously reported mutant strains (Δ*hapX,* Δ*srbA*). Furthermore, the frequently used wild-type strain Af293 showed largely decreased and delayed growth in liquid shake cultures compared to other strains (AfS77, A1160p+, AfS35). Taken together, the CGQ allows for robust, automated biomass monitoring of *A. fumigatus* during liquid shake conditions, which largely facilitates the characterization of the growth pattern of filamentous fungal species.

## 1. Introduction

Filamentous fungi are ubiquitously found in nature as they are capable of adapting to diverse environments. Some species such as *Aspergillus nidulans* and *Neurospora crassa* serve as model organisms to study biological processes, others such as *Aspergillus niger*, *Aspergillus oryzae*, *Penicillium chrysogenum*, and *Trichoderma reesei* are used extensively as workhorses in biotechnology, and numerous others are pathogens for plants, animals and/or humans or employed for biocontrol [1,2,3,4,5,6]. Growth studies, for example to compare wild-type and mutant strains or to elucidate the response to different nutrients, are mainly carried out on solid media. However, corresponding studies concerning gene expression as well as biochemical or metabolic aspects are carried out usually in liquid shake conditions, which do not correspond to the growth pattern on solid media. The reason for this practice is that, in contrast to yeast and bacterial cells, most filamentous fungi form pellets in liquid shake cultures, which hinders growth determination by classical optical density measurement such as at 600 nm (OD_600_). The major solution to this is usually the time-consuming and tedious process of dry-weight (DW) determination, which is lacking in most studies. Rarely, DW determination in liquid shake cultures from a single time point is performed, which still does not reveal growth phase details.

However, culturing on solid and in liquid media differs in oxygen supply, nutrient contact, and shear forces, which affect not only metabolism but also morphology, differentiation, and development, e.g., most fungal species such as *Aspergillus fumigatus* or *A. nidulans* lack conidiation in liquid shake conditions (e.g., [7]). In contrast to bacteria and yeast, culturing of filamentous fungal species usually starts with dormant conidia, which differ in cell wall morphology from hyphae [8,9,10]. The outer layer of *A. fumigatus* conidia, termed the rodlet layer, is composed of a hydrophobic polymerized hydrophobin and is underlaid by melanin. Underneath, the cell wall is composed mainly of polysaccharides such as glucan, chitin, mannan, and galactosaminogalactan. The outermost layer of hyphae is composed of alpha-glucan, which covers the beta-1,3-glucan and chitin layers. Germination starts with isotropic growth that involves water uptake and cell wall growth (termed swelling) followed by polarized growth that results in the formation of a germ tube from which the new mycelium originates [11,12]. During the hyphal growth stage, filamentous fungi secrete an extracellular matrix composed mainly of proteins, lipids, and polysaccharides such as galactosaminogalactan, α-glucan, and galactomannan [13,14]. Surface exposure of adhesive α-glucan and galactosaminogalactan during germination is believed to cause hyphal aggregation that is responsible for the pellet formation usually observed in liquid shake cultures [15,16]. The pellet formation causes the heterogenous growth of filamentous fungal species, as the surface and the interior of pellets differ significantly with respect to supply of nutrients and oxygen [17]. Recently, impairment of biosynthesis of α-glucan and galactosaminogalactan was shown to lead to dispersed hyphal growth facilitating biomass determination via optical density [16] but these genetic modifications severely affect the physiology.

**Figure 1:**
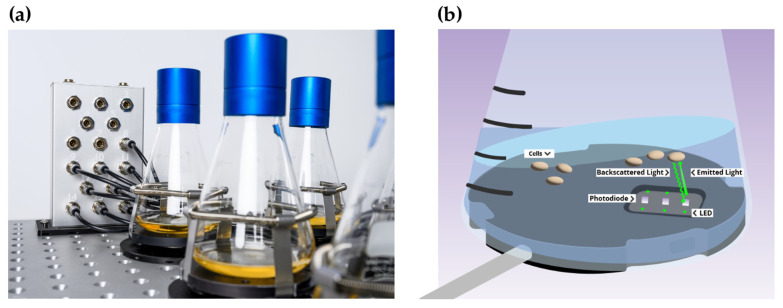
The CGQ-measurement principle. (**a**) CGQ sensors are mounted underneath the shake flasks for non-invasive measurements through the glass wall of the vessel. The sensors are connected to a base station that communicates the data to a computer outside of the incubator. (**b**) Biomass measurements are mediated via backscattered light detection. An LED emits light into the medium, which is scattered by cells/pellets/particles within the culture. A portion of the scattered light is detected as backscatter by a photodiode that is part of the CGQ sensor. With higher cell densities the backscattered light intensity is higher compared to lower cell densities.

Using the facultative pathogenic mold *A. fumigatus* as an example [18], we here evaluated the applicability of the CGQ (“cell growth quantifier”) by Scientific Bioprocessing (SBI), previously Aquilabiolabs, for automated, non-invasive, and parallelizable online-monitoring of biomass formation of filamentous fungi in liquid shake cultures. Biomass monitoring by the CGQ is realized by backscattered light measurements. For this, a sensor plate comprising an LED light source and a photodiode is mounted into the spring clamp of the shaker. Through a wire, it is connected with a base station that supplies power to the sensor plates and communicates acquired data to a computer (Figure 1a). This set-up allows measurement from the bottom of the Erlenmeyer flask positioned on top of the sensor (Figure 1b). The LED emits light with a central wavelength of 520 nm into the cultivation broth, of which a certain percentage is scattered back by particles and cells in the medium (Figure 1b). The higher the cell density in the medium, the more light is scattered back and detected by the photodiode which converts the photons into a weak electric current that is subsequently amplified and digitized, yielding a single backscatter reading. Up to a million of backscatter readings are collected within one measurement cycle of 1–2 s at a dynamic measurement frequency (>500 kHz), resulting in a high raw data density and high resolution of one data point. These periodic raw signal series represent a complete image of the dynamic liquid distribution within several shaking movements, factoring in, e.g., different liquid heights above the sensor and irregular signals due to non-homogenous cell suspensions [19]. This high-speed data acquisition therefore allows to smooth out the heterologous distribution of cells in cultures with filamentously growing organisms, in contrast to approaches with single static measurements. Furthermore, the automated set-up combined with real-time data output offers convenient monitoring even for prolonged fermentation times and enables the immediate detection of relevant growth events. Visualization, data analysis, and comparison can be carried out using the provided software. The CGQ allows fully parallelized biomass monitoring of up to 16 flasks, all connected to one base station installable in standard shaking incubators. The fully automated measurements under continuous shaking are favorable to avoid effects such as sedimentation, poor aeration and mixing, anaerobic metabolic stress, and to reduce the scientist’s manual workload.

To evaluate the applicability of CGQ, we compared CGQ-mediated growth monitoring with that of gravimetric dry-weight determination from certain time points including four frequently used *A. fumigatus* wild-type strains (AfS77, A1160p+, AfS35, Af293), mutants with previously reported growth defects (Δ*hapX*, Δ*srbA*), and under the limitation of iron and nitrogen.

## 2. Materials and Methods

### 2.1. Fungal Strains and Growth Conditions

The used *A. fumigatus* strains were AfS77 (a Ku70-lacking derivative of the clinical isolate ATCC46645 [20]), the AfS77-derived Δ*hapX* and Δ*srbA* mutant strains [21,22], AfS35 (a Ku70-lacking derivative of the clinical isolate D141 [23]), A1160p+ (a Ku80-lacking derivative of the clinical isolate CEA10 [24]), and the clinical isolate Af293 [25]. The strains were grown at 37 °C on solid or in liquid Aspergillus minimal media (AMM) according to Pontecorvo et al. [26] with 1% glucose as carbon source and, if not stated otherwise, 20 mM glutamine as the nitrogen source with a pH set to 6.5. Iron replete media contained 0.03 mM FeSO_4_ as the iron source; for iron starvation, the addition of iron was omitted. For plate growth assays, AMM was solidified by the addition of 1.5% agar (Oxoid bacteriological agar, LP0011), and 1 × 10^4^ conidia were point-inoculated and plates were incubated for 48 h at 37 °C. For all liquid shake cultures, 100 mL AMM in 0.5 L Erlenmeyer flasks were inoculated with 10^6^/mL conidia followed by incubation in an Infors HT Multitron Standard incubation shaker at 37 °C with 200 rpm for the time indicated. For CGQ-mediated growth measurements, six flasks connected to a single base station were monitored in parallel, which allowed to include two experiments, each with biological triplicates, in the same run. At the end of each experiment, mycelia was harvested by filtration and DW determined after freeze drying. Moreover, the pH of the supernatant was recorded routinely and the protease activity occasionally.

### 2.2. Determination of Protease Activity in Culture Supernatant

For semi-quantitative determination of proteolytic activity, an assay based on clearance of unprocessed X-ray film material was applied [27,28]. Therefore, 8 µL aliquots of serial two-fold dilution series (1:1–1:16) of culture supernatants in phosphate buffered saline (PBS, pH 7.4) were spotted onto sheets of unprocessed X-ray films and incubated for 45 min at 37 °C in a humid chamber. Gelatin hydrolysis in the light-sensitive layer causes a clearing zone, which is indicative for protease activity.

### 2.3. Determination of Pellet Morphology and Size Distribution

Fungal pellets were removed from liquid shake cultures and 2 mL of culture were diluted 1:10 in water in a 9.6 cm petri dish. For size determination, pictures were taken with a Nikon D700 camera and a Tamron SP Macro lens mounted onto a stand at fixed distance to allow for comparison of different petri dishes. Following the import and black/white conversion of pictures, fungal pellets were quantified using a modified custom ImageJ macro with the watershed algorithm [29,30]. For plotting, resulting particle areas were filtered for a circularity of ≥0.75. Microscopic images of pellets were acquired by bright field microscopy at 40× magnification.

### 2.4. Presentation of CGQ-Mediated Growth Monitoring and Statistics

Raw data were exported as MS Excel spreadsheets with 60 s intervals and further processed for plotting in R using the packages tidyverse, ggplot2, ggpubr, and openxlsx [31,32,33,34,35]. The background of individual runs was corrected for by subtracting 98% of the mean of the first 200 data points (i.e., the first 3.3 h of monitoring) from the measured backscatter (BS) values. The biological triplicates of each experiment were plotted as lines. To better illustrate growth dynamics of tested conditions, smoothed conditional means of the respective replicates were included as well. Smoothing occurred by local polynomial regression fitting using the LOESS algorithm with a span value of a = 0.3. To relate final BS values to the dry-weight (DW) of a run, the mean of the last 15 BS data points of each run was used. The statistical significance of pH, DW, and final BS values was calculated in R by one-way ANOVA and Tukey’s multiple comparison test.

## 3. Results and Discussion

### 3.1. CGQ Characterizes the Growth Curve of A. fumigatus during Iron Sufficiency and Iron Starvation

**Figure 2:**
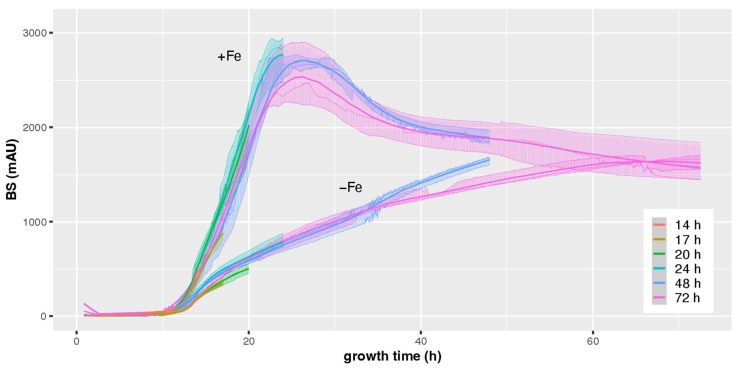
CGQ-mediated growth monitoring of *A. fumigatus* AfS77 during 14–72 h liquid shake culturing under +Fe and −Fe conditions. *A. fumigatus* AfS77 was cultured for 14 h, 17 h, 20 h, 24 h, 48 h, and 72 h in liquid shake cultures under −Fe and +Fe conditions in biological triplicates and biomass was monitored with the CGQ. The different colors discriminate the cultivations conducted for different incubation times. Thin lines display growth dynamics of individual runs and shaded areas show the variance within the parallels of each experiment. Thick lines represent a smooth curve fitted by local polynomial regression using the LOESS algorithm with a span value of a = 0.3.

To evaluate the general validity of CGQ-derived online biomass monitoring of *A. fumigatus*, strain AfS77 was grown in biological triplicates for 14 h, 17 h, 20 h, 24 h, 48 h, and 72 h at 37 °C in iron-replete (+Fe) or iron depleted (−Fe) minimal medium. This approach was chosen to be able to monitor dry weight (DW) at different time points over 72 h of cultivation. All cultures were CGQ recorded and at the end of the cultivation the DW and pH was determined (see Tables S1 and S2 for raw data). Media were inoculated with dormant conidia, which display swelling at about four hours and germination at about seven hours after inoculation in this liquid medium [36]. In agreement, backscatter (BS) measurements did not indicate any increase in biomass formation in the first 10 h of cultivation; subsequently CGQ-mediated measurement indicated a fast increase of biomass in both +Fe and −Fe conditions (Figure 2). In +Fe conditions, biomass formation peaked at about 24 h and subsequently decreased. Notably, from a starting pH of 6.5, the +Fe cultures displayed initial acidification of growth medium to a pH of 3.6 at the 20 h time point with subsequent alkalinization to a pH of 5.9 at 24 h and 8.6 at 48 h and 72 h (Table 1).

**Figure 3:**
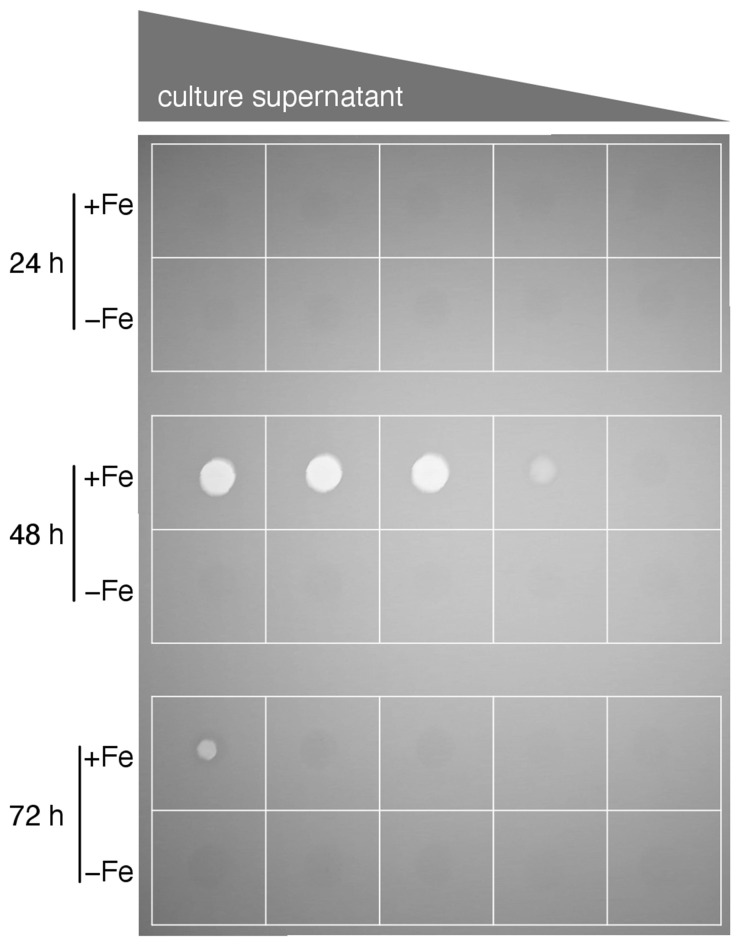
Protease activity is detectable in culture supernatants of +Fe cultures at 48 h and to a lesser extent in 72 h but not in those of younger (24 h) +Fe or in −Fe cultures. Supernatants from cultures were spotted onto an unprocessed X-ray film to test for the presence of proteolytic activities that hydrolyze the gelatin-containing light-sensitive layer (visible as bright circles). Two-fold dilution series (1:1–1:16) in PBS is indicated on top of the picture. The decrease of proteolytic activity in 72 h +Fe compared to 48 h +Fe cultures is most likely caused by degradation of the proteases.

Concomitant with the alkaline pH of the culture supernatant, protease activity was detected in culture supernatants at 48 h and 72 h (Figure 3). This pattern is consistent with autolytic processes at 48 h and 72 h time points [37] which is in agreement with the decrease in biomass in this culture phase. In contrast to the +Fe cultures, BS measurement indicated a continuous increase in biomass in the −Fe cultures, however, at a significant lower level compared to the +Fe cultures as expected from this growth-limiting condition. In contrast to +Fe cultures, the −Fe cultures displayed continuous acidification and a lack of detectable protease activity, which is in agreement with a lack of autolysis.

**Figure 4:**
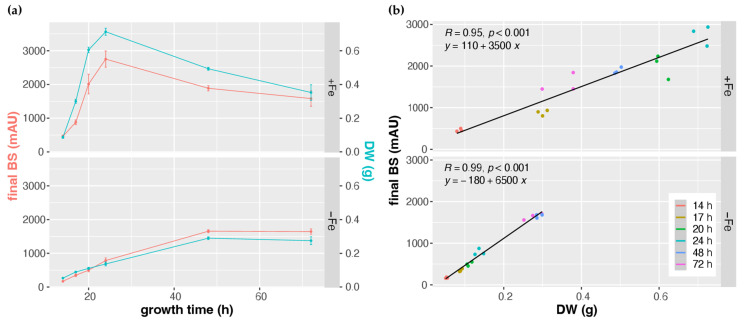
Positive correlation of CGQ-mediated growth monitoring and classical biomass measurement of *A. fumigatus* AfS77 during 14–72 h liquid shake culturing under +Fe and −Fe conditions. Data were taken from the experiments described in Figure 2 and Table 1**.** (**a**) Time course graphs of mean final backscatter (final BS) and mean dry-weight (DW) ± standard deviation of 3 runs each. (**b**) Final BS plotted vs. DW with colored dots representing endpoints of individual cultures grown for the times indicated. Pearson correlation coefficient and corresponding *p*-values are displayed in the upper left side of the panels. Regression lines and corresponding equations based on linear models fitted to the scatter plots are shown.

The online BS measurements largely matched the offline DW determination in +Fe and −Fe cultures, i.e., it portrayed the growth curves during +Fe and −Fe conditions as well as the decreased biomass formation in −Fe compared to +Fe conditions (Table 1 and Figure 4a). However, the observed BS/DW ratios shown in Table 1 varied up to a factor of about 2 (2932 from 17 h +Fe and 5995 of 72 h −Fe). For example, the BS/DW ratio was higher at the 14 h and at the 72 h time points compared to the other time points (17 h, 20 h, 24 h, 48 h) in +Fe cultures. Moreover, starting at 20 h, the BS/DW ratio was significantly higher in −Fe compared to +Fe conditions. Taken together, these data indicate that the BS measurements are directly proportional to the DW, however, with slopes depending on the growth condition. To give an example: similar to the 17 h +Fe DW with 0.30 g, the 48 h −Fe DW was 0.29 g, while the BS measurements were 880 ± 55 and 1656 ± 36 in +Fe and −Fe conditions, respectively. These results indicate that factors other than the biomass impacted the measurement.

**Figure 5:**
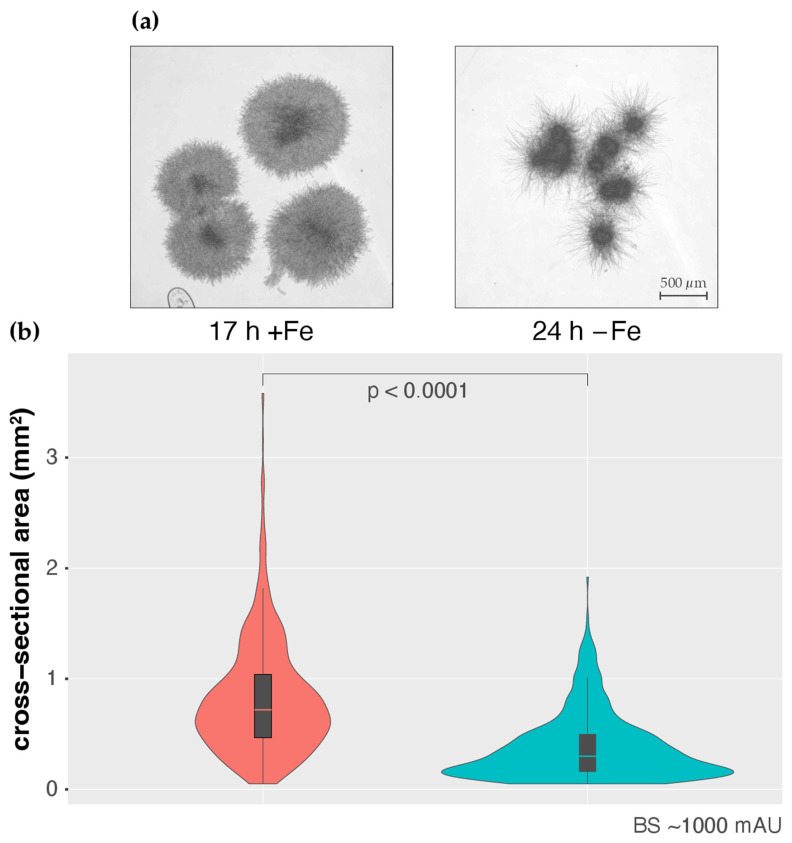
Pellet morphology differs in +Fe and −Fe conditions. (**a**) *A. fumigatus* AfS77 pellets were removed for microscopic analysis when base-line-corrected BS reached values of approximately 1000 mAU, after growth times of 17 h and 24 h for +Fe and −Fe conditions, respectively. (**b**) Violin plots showing the distribution of pellet cross-sectional area in +Fe and −Fe cultures at BS~1000 mAU. Box plots showing medians (0.72 mm^2^ and 0.30 mm^2^ for +Fe and −Fe, respectively), interquartile ranges, and spikes extending to the upper- and lower-adjacent values in each group are overlaid. Outliers are not shown.

To analyze this, *A. fumigatus* strain AfS77 was cultured to a BS value of about 1000 under both +Fe (17 h) and −Fe (24 h). As shown in Figure 5, the pellet morphologies were found to significantly differ in +Fe and −Fe conditions, with pellets being smaller but significantly more lacerated in −Fe compared to +Fe conditions (Figure 5a). These data indicate that the pellet morphology impacts the BS measurement, which is expected from an optics-based system. Indeed, as inferred from the Mie theory of scattering, there is a negative correlation of light scattering and particle volume of spherical objects [38], indicating that the smaller fungal pellets, as observed under −Fe conditions, exhibit relatively higher backscattering. Nevertheless, BS measurements and DW showed high Pearson correlation coefficients of 0.95 and 0.99 in +Fe and −Fe conditions, respectively (Figure 4b), indicating a good overall correlation. However, the correlation factor (regression line and corresponding equation) depends on the growth conditions, i.e., +Fe or −Fe conditions (Figure 4b), most likely due to different pellet morphology (Figure 5a).

### 3.2. CGQ Provides Reproducible Growth Curves

**Figure 6:**
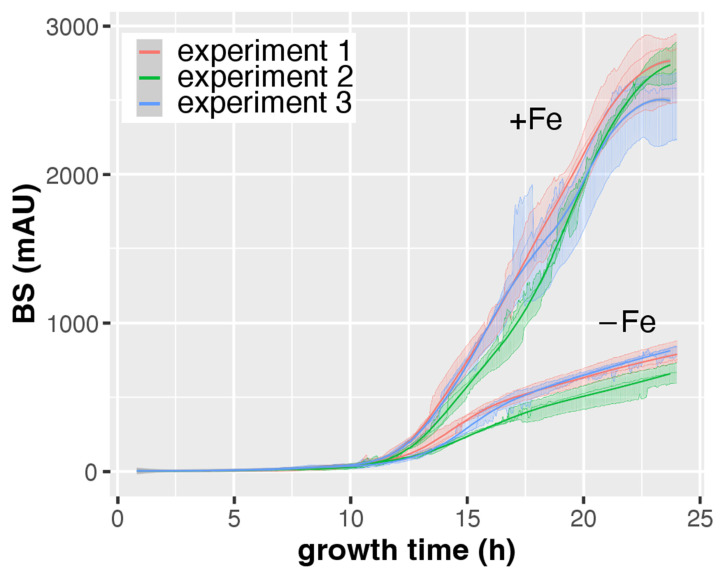
CGQ-mediated growth monitoring of *A. fumigatus* AfS77 cultured for 24 h at different days (experiments 1–3) under +Fe and −Fe conditions. Each experiment included biological triplicates. Details of the graph are as in the caption of Figure 2.

As shown in Figure 2, the growth curves of the 14 h, 17 h, 20 h, 24 h, 48 h, and 72 h cultures are highly similar in the overlapping time frames, which indicates high reliability of individual CGQ-mediated growth measurements. To further investigate robustness of CGQ, *A. fumigatus* AfS77 was grown in triplicates in three experiments at different days for 24 h under +Fe and −Fe conditions. As seen in Figure 6 and Table 2, the growth curves observed in the different experiments were highly similar, underlining that CGQ-mediated growth monitoring is highly reproducible.

### 3.3. CGQ Portrays Growth Differences due to Nitrogen Limitation

**Figure 7:**
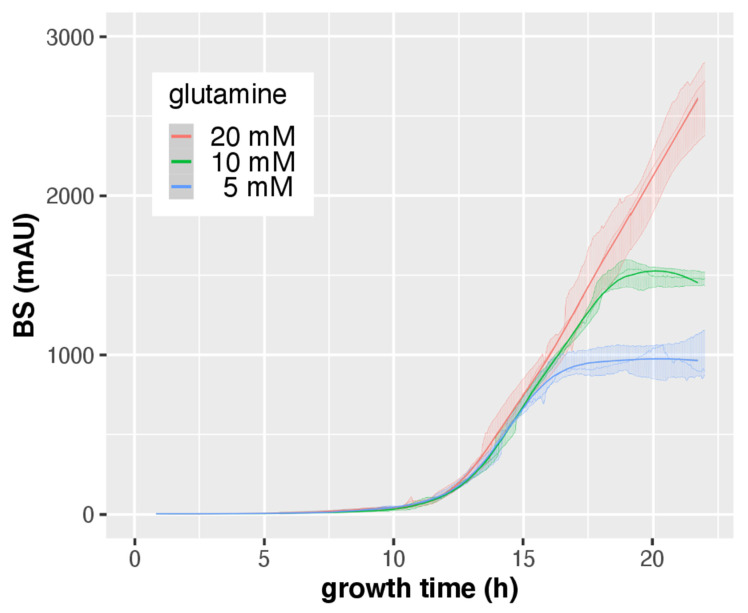
CGQ-mediated growth monitoring of *A. fumigatus* AfS77 cultured in biological triplicates for 22 h with 20 mM, 10 mM, or 5 mM glutamine (Gln). Details of the graph are as in the caption of Figure 2.

In a next step, the growth of *A. fumigatus* AfS77 in media with different nitrogen availability was compared. Therefore, *A. fumigatus* was cultured at 37 °C in the presence of 20 mM, 10 mM, or 5 mM glutamine for 24 h. BS measurements indicated that 10 mM and particularly 5 mM glutamine significantly reduced biomass formation (Figure 7), which was confirmed by DW determination (Table 3). Together with the comparison of growth during +Fe and −Fe conditions (Figure 2), these data demonstrate that CGQ can be used for the characterization of growth media such as the identification of growth-limiting nutrient concentrations. Similar to iron starvation, nitrogen starvation increased the BS/DW ratio, indicating that starvation alters pellet morphology.

### 3.4. CGQ Delineates Growth Defects of A. fumigatus Mutant Strains Lacking Either HapX or SrbA

**Figure 8:**
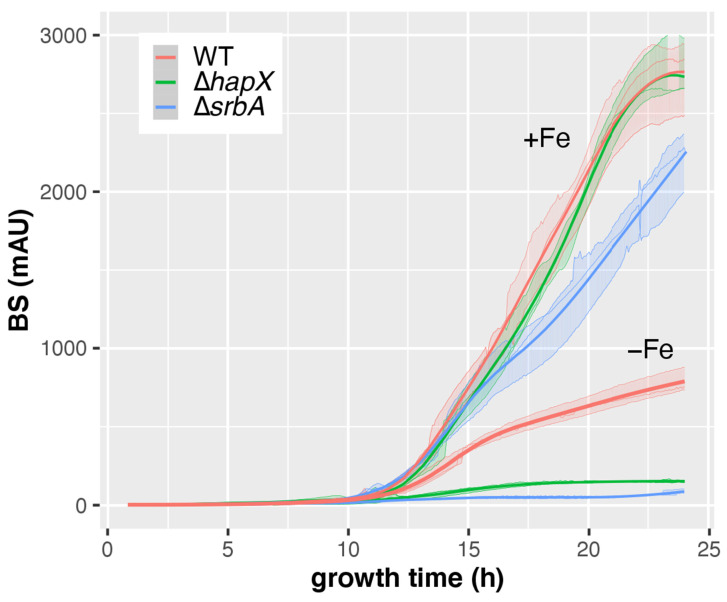
CGQ-mediated growth monitoring of *A. fumigatus* AfS77 (WT) compared to Δ*hapX* and Δ*srbA* mutant strains cultured in biological triplicates for 24 h under +Fe and −Fe conditions. Details of the graph are as in the caption of Figure 2.

To evaluate the applicability of CGQ for detecting growth defects of mutant strains, the AfS77-derived mutants Δ*hapX* and Δ*srbA* were employed [21,22]. Δ*hapX* lacks the iron-regulatory bZIP transcription factor HapX and Δ*srbA* lacks the sterol regulatory element binding protein (SREBP) SrbA. As revealed previously [21,22] and confirmed here by single time point DW measurements after liquid shake cultivation (Table 4), Δ*hapX* shows wild-type-like biomass formation in +Fe conditions but a significant growth defect under −Fe conditions, while Δ*srbA* displays a mild growth defect under +Fe conditions and a more severe growth defect than Δ*hapX* under −Fe conditions. The growth defects of Δ*hapX* and Δ*srbA*, as well as the growth differences between Δ*hapX* and Δ*srbA*, were clearly detected by BS measurements (Figure 8). These data demonstrate that CGQ visualizes growth pattern differences of mutant strains.

**Figure 9:**
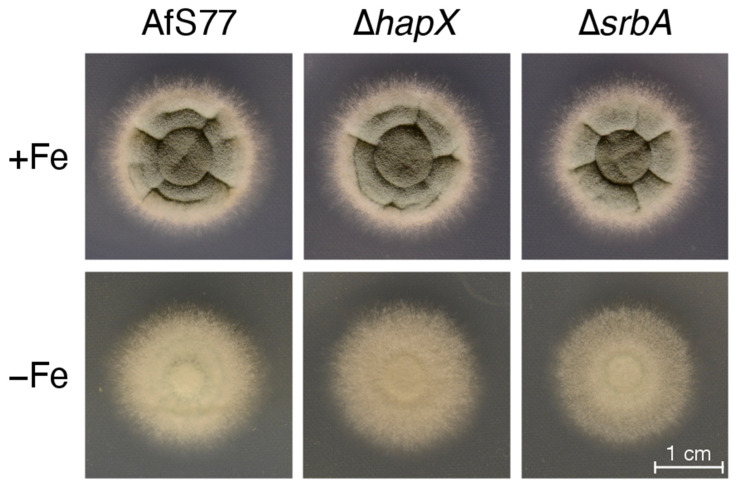
Colony morphology of *A. fumigatus* AfS77 compared to ∆*hapX* and ∆*srbA* cultured for 48 h at 37 °C under +Fe and −Fe conditions. For inoculation of fungal strains, suspensions containing 10^4^ spores were dotted onto minimal media solidified with 1.5% agar.

For comparison, *A. fumigatus* AfS77, Δ*hapX*, and Δ*srbA* strains were grown on solid minimal medium; i.e., the same medium of the liquid culture (Figure 8) was used in solidified form by adding 1.5% agar (Figure 9). Compared to +Fe conditions, the strains on −Fe conditions showed lower pigmentation, reflecting decreased sporulation. In contrast to biomass formation in liquid shake conditions (Figure 8 and Table 4), however, the radial growth of all three strains was largely similar. These data emphasize that growth on solid media does not reflect behavior in liquid shake conditions. The most likely explanations are that the significantly higher biomass formation in liquid shake conditions increases iron limitation by iron consumption and that liquid shake culturing decreases the oxygen supply, which might play a particular role for the Δ*srbA* mutant, which shows a hypoxic growth defect [22].

### 3.5. CGQ Displays Differences in Growth of Different A. fumigatus Laboratory Type Strains

*A. fumigatus* is a ubiquitous environmental mold and the leading cause of diverse human diseases ranging from allergenic bronchopulmonary aspergillosis to invasive pulmonary aspergillosis [18]. Experimental investigations of the biology and virulence of this opportunistic pathogen have historically been based on the use of a few type strains [24] including AfS77 (a Ku70-lacking derivative of the clinical isolate ATCC46645 [20]), AfS35 (a Ku70-lacking derivative of the clinical isolate D141 [23]), A1160p+ (a Ku80-lacking derivative of the clinical isolate CEA10 [24]), and the clinical isolate Af293 [25]. Notably, Af293 was the first *A. fumigatus* strain with a resolved and annotated genome [25]. However, several studies revealed significant differences in physiological responses to abiotic stimuli and virulence in murine models of invasive pulmonary aspergillosis between *A. fumigatus* Af293 and other *A. fumigatus* isolates including commonly used *A. fumigatus* CEA10 [39,40]. Due to severely decreased growth of Af293 compared to other strains in a minimal medium broth, Sugui et al. [41] concluded that Af293 possesses a nutritional deficiency and Kowalski et al. [42] demonstrated decreased fitness during hypoxic conditions such as in liquid shake conditions. In a next step, the growth of these four commonly used strains employing CGQ-mediated growth monitoring was compared.

**Figure 10:**
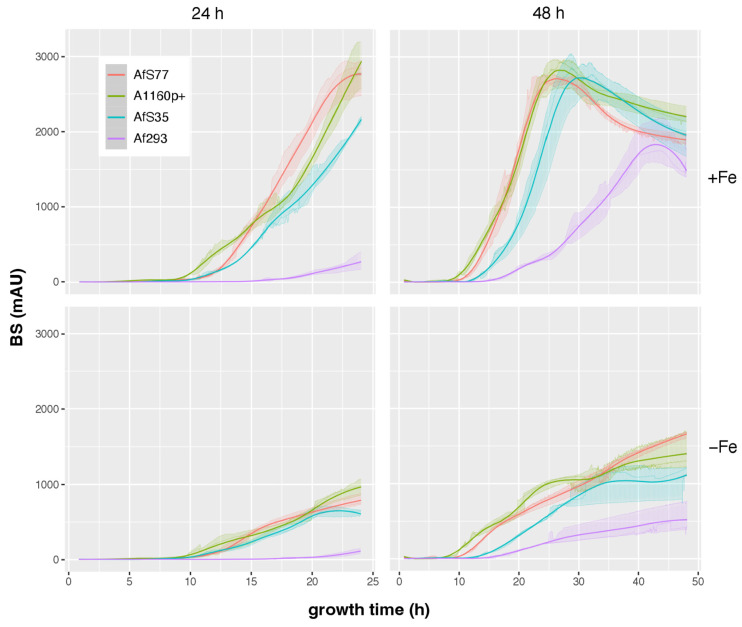
CGQ-mediated growth monitoring of *A. fumigatus* AfS77 compared to A1160p+, AfS35, and Af293 cultured for 24 h and 48 h under +Fe and −Fe conditions. Details of the graph are as in the caption of Figure 2.

In agreement with single time point DW measurements after liquid shake cultivation for 24 h under both +Fe and −Fe conditions (Table 5), BS measurements demonstrated negligible growth of Af293 compared to the other three strains, which displayed largely similar growth patterns (Figure 10). Growth monitoring by BS measurement and DW for 48 h revealed that growth of Af293 is not generally poor in this minimal medium but that the maximal growth is delayed, peaking at about 42 h in +Fe conditions, a time point at which all three other strains are already in the autolytic phase (Figure 10 and Table 5). These data emphasize the value of CGQ-mediated growth monitoring, as this liquid shake growth behavior of Af293 has not been observed previously because it is extremely difficult to monitor with classical methods. Both BS measurements and DW determination indicated that AfS35 displays slightly delayed biomass formation during +Fe conditions (Figure 10). Taken together, CGQ-mediated growth monitoring elucidated growth pattern differences of widely used laboratory type strains.

**Figure 11:**
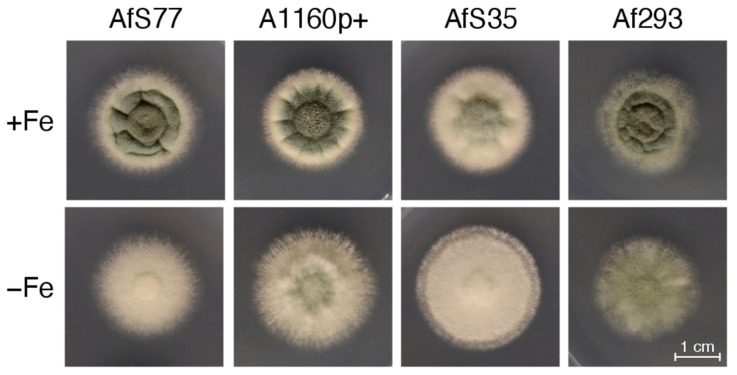
Colony morphology of *A. fumigatus* AfS77 compared to A1160p+, AfS35, and Af293 cultured for 48 h at 37 °C under +Fe and −Fe conditions. For inoculation of fungal strains, suspensions containing 10^4^ spores were dotted onto minimal media solidified with 1.5% agar.

For comparison, *A. fumigatus* AfS77, AfS35, A1160p+, and Af293 were grown on solid minimal medium; i.e., the very same medium solidified with 1.5% agar was used (Figure 11). In contrast to biomass formation in liquid shake conditions (Figure 10 and Table 5), however, Af293 displayed a similar radial growth on solid medium. These data emphasize again that growth on solid media might not reflect behavior in liquid shake conditions. The most likely explanation is the previously described decreased fitness of Af293 during hypoxic conditions [42].

## 4. Conclusions

CGQ-mediated online growth monitoring allowed the robust characterization of the growth curve of *A. fumigatus* including the autolytic phase as shown by comparison of online BS measurements and offline DW determinations at certain time intervals. BS measurements and DW showed high Pearson correlation coefficients. However, the ratio between BS measurements and DW depended on the strain and the growth conditions such as +Fe or −Fe, most likely due to different pellet morphologies found under these conditions. CGQ-mediated online growth monitoring revealed growth differences due to the limitations of nutrients such as iron or nitrogen, growth defects caused by gene defects such as a lack of SrbA or HapX, and growth defects of a commonly used laboratory type strain. Taken together, CGQ represents a valuable new tool for the growth curve characterization of filamentous fungal species, strains, and mutants as well as analyzing media compositions.

## Figures and Tables

**Table 1:** Comparison of DW, BS values, BS/DW ratios, and pH values of *A. fumigatus* AfS77 cultured for 14–72 h under +Fe and −Fe conditions. Mean ± standard deviation of biological triplicates is displayed.

		14 h	17 h	20 h	24 h	48 h	72 h
+Fe	pH ± SD	5.69 ± 0	3.99 ± 0.04	3.62 ± 0.03	5.9 ± 0.44	8.63 ± 0.01	8.72 ± 0.05
	DW ± SD (g)	0.09 ± 0.01	0.3 ± 0.01	0.6 ± 0.02	0.71 ± 0.02	0.49 ± 0.01	0.35 ± 0.05
	final BS ± SD	461 ± 36	880 ± 68	2011 ± 294	2752 ± 240	1887 ± 78	1582 ± 227
	BS/DW ± SD	5328 ± 369	2932 ± 227	3334 ± 564	3865 ± 379	3826 ± 92	4515 ± 590
−Fe	pH ± SD	5.83 ± 0.02	5.27 ± 0.08	4.79 ± 0.06	4.13 ± 0.06	4.04 ± 0.03	4.04 ± 0.03
	DW ± SD (g)	0.05 ± 0	0.09 ± 0	0.11 ± 0.01	0.14 ± 0.01	0.29 ± 0.01	0.27 ± 0.02
	final BS ± SD	170 ± 13	348 ± 37	500 ± 49	786 ± 78	1656 ± 44	1645 ± 78
	BS/DW ± SD	3234 ± 261	3896 ± 269	4514 ± 286	5763 ± 681	5718 ± 164	5995 ± 231

**Table 2:** Comparison of DW, BS values, BS/DW ratios, and pH values of *A. fumigatus* AfS77 cultured for 24 h at different days under +Fe and −Fe conditions. Mean ± standard deviation of biological triplicates is displayed. Statistically significant differences (marked by ^a^) of −Fe compared to +Fe conditions were assessed by one-way ANOVA and Tukey’s multiple comparison test.

		Experiment 1	Experiment 2	Experiment 3
+Fe	pH ± SD	5.9 ± 0.44	5.63 ± 0.25	6.24 ± 0.1
	DW ± SD (g)	0.71 ± 0.02	0.73 ± 0.01	0.73 ± 0.01
	final BS ± SD	2752 ± 240	2727 ± 132	2496 ± 236
	BS/DW ± SD	3865 ± 379	3752 ± 189	3415 ± 319
−Fe	pH ± SD	4.13 ± 0.06 ^a^	4 ± 0 ^a^	4.43 ± 0.1 ^a^
	DW ± SD (g)	0.14 ± 0.01 ^a^	0.11 ± 0.01 ^a^	0.14 ± 0.01 ^a^
	final BS ± SD	786 ± 78 ^a^	662 ± 66 ^a^	815 ± 41 ^a^
	BS/DW± SD	5763 ± 681	5848 ± 1079	5837 ± 368

^a^ *p* < 0.001.

**Table 3:** Comparison of DW, BS values, BS/DW ratios, and pH values of *A. fumigatus* AfS77 cultured in the presence of different glutamine (Gln) concentrations for 24 h. Mean ± standard deviation of biological triplicates is displayed. Statistically significant differences (marked by ^a^ or ^b^) of Gln limitation (5 mM and 10 mM) compared to the standard concentration (20 mM) were assessed by one-way ANOVA and Tukey’s multiple comparison test.

	Gln Concentration		
	20 mM	10 mM	5 mM
pH ± SD	5.9 ± 0.44	6.93 ± 0.06 ^a^	6.83 ± 0.06 ^b^
DW ± SD (g)	0.71 ± 0.02	0.35 ± 0.01 ^b^	0.16 ± 0 ^b^
final BS ± SD	2752 ± 240	1477 ± 40 ^b^	1052 ± 252 ^b^
BS/DW ± SD	3865 ± 379	4237 ± 30	6523 ± 1657

^a^ *p* < 0.01, ^b^ *p* < 0.001.

**Table 4:** DW, BS values, BS/DW ratios, and pH values of *A. fumigatus* AfS77 compared the Δ*hapX* and Δ*srbA* mutant strains cultured for 24 h under +Fe and −Fe conditions. Mean ± standard deviation of biological triplicates is displayed. Statistically significant differences (marked by ^a^, ^b^, or ^c^) of mutant strains compared to AfS77 were assessed by one-way ANOVA and Tukey’s multiple comparison test.

		AfS77	∆*hapX*	∆*srbA*
+Fe	pH ± SD	5.9 ± 0.44	5.43 ± 0.59	4.17 ± 0.06 ^c^
	DW ± SD (g)	0.71 ± 0.02	0.71 ± 0	0.53 ± 0.03 ^c^
	final BS ± SD	2752 ± 240	2769 ± 192	2197 ± 196 ^a^
	BS/DW ± SD	3865 ± 379	3898 ± 256	4102 ± 146
−Fe	pH ± SD	4.13 ± 0.06	4.2 ± 0.1	5.9 ± 0
	DW ± SD (g)	0.14 ± 0.01	0.06 ± 0.01 ^c^	0.02 ± 0 ^c^
	final BS ± SD	786 ± 78	153 ± 6 ^c^	89 ± 14 ^b^
	BS/DW ± SD	5763 ± 681	2594 ± 328	3573 ± 523

^a^ *p* < 0.05, ^b^ *p* < 0.01, ^c^ *p* < 0.001.

**Table 5:** Comparison of DW, BS values, BS/DW ratios, and pH values of *A. fumigatus* AfS77 and A1160p+, AfS35, and Af293 cultured for 24 h and 48 h under +Fe and −Fe conditions. Mean ± standard deviation of biological triplicates is displayed. Statistically significant differences (marked by ^a^, ^b^, or ^c^) of strains compared to AfS77 were assessed by one-way ANOVA and Tukey’s multiple comparison test.

			AfS77	A1160p+	AfS35	Af293
24 h	+Fe	pH ± SD	5.9 ± 0.44	4.9 ± 0.26	3.5 ± 0.06	5.65 ± 0.31
		DW ± SD (g)	0.71 ± 0.02	0.69 ± 0 ^c^	0.6 ± 0.03 ^c^	0.04 ± 0.01 ^c^
		final BS ± SD	2752 ± 240	2845 ± 322 ^c^	2146 ± 47 ^b^	271 ± 116 ^c^
		BS/DW ± SD	3865 ± 379	4119 ± 483	3596 ± 229	6088 ± 1507
	−Fe	pH ± SD	4.13 ± 0.06	3.5 ± 0 ^a^	3.82 ± 0.08	6.09 ± 0.08 ^c^
		DW ± SD (g)	0.14 ± 0.01	0.09 ± 0.01 ^a^	0.11 ± 0	NA ^d^
		final BS ± SD	786 ± 78	964 ± 105	631 ± 42	109 ± 35 ^b^
		BS/DW ± SD	5763 ± 681	10,990 ± 1862	5696 ± 411	NA ^d^
48 h	+Fe	pH ± SD	8.63 ± 0.01	8.76 ± 0.04	8.68 ± 0.06	6.71 ± 0.11 ^c^
		DW ± SD (g)	0.49 ± 0.01	0.39 ± 0.01 ^c^	0.48 ± 0.02	0.62 ± 0.03 ^c^
		final BS ± SD	1887 ± 78	2207 ± 116	1962 ± 263	1583 ± 203
		BS/DW ± SD	3826 ± 92	5613 ± 342	4130 ± 678	2538 ± 280
	−Fe	pH ± SD	4.04 ± 0.03	4.12 ± 0.02	3.45 ± 0.03 ^c^	5.23 ± 0.2 ^c^
		DW ± SD (g)	0.29 ± 0.01	0.17 ± 0.01 ^c^	0.18 ± 0.01 ^c^	0.11 ± 0.02 ^c^
		final BS ± SD	1656 ± 44	1404 ± 247	1172 ± 62	515 ± 208 ^c^
		BS/DW ± SD	5718 ± 164	8330 ± 1954	6644 ± 388	4422 ± 952

^a^ *p* < 0.05, ^b^ *p* < 0.01, ^c^ *p* < 0.001, ^d^ NA: biomass was too low for DW determination.

## Data Availability

Data is contained within the article or Supplementary Materials.

## Supplementary Materials

The following supporting information can be downloaded at: https://www.mdpi.com/article/10.3390/jof8101013/s1, Table S1: BS_raw_data.xlsx; Table S2: DW_pH_raw_data.xlsx.

## References

[1] Meyer V., Andersen M.R., Brakhage A.A., Braus G.H., Caddick M.X., Cairns T.C., de Vries R.P., Haarmann T., Hansen K., Hertz-Fowler C. (2016). Current challenges of research on filamentous fungi in relation to human welfare and a sustainable bio-economy: A white paper. Fungal Biol. Biotechnol..

[2] Yu Y., Hube B., Kämper J., Meyer V., Krappmann S. (2017). When green and red mycology meet: Impressions from an interdisciplinary forum on virulence mechanisms of phyto- and human-pathogenic fungi. Virulence.

[3] Brown G.D., Denning D.W., Gow N.A.R., Levitz S.M., Netea M.G., White T.C. (2012). Hidden killers: Human fungal infections. Sci. Transl. Med..

[4] Behera B.C. (2020). Citric acid from *Aspergillus niger*: A comprehensive overview. Crit. Rev. Microbiol..

[5] Bischof R.H., Ramoni J., Seiboth B. (2016). Cellulases and beyond: The first 70 years of the enzyme producer *Trichoderma reesei*. Microb. Cell Fact..

[6] Schmoll M., Dattenböck C., Carreras-Villaseñor N., Mendoza-Mendoza A., Tisch D., Alemán M.I., Baker S.E., Brown C., Cervantes-Badillo M.G., Cetz-Chel J. (2016). The Genomes of Three Uneven Siblings: Footprints of the Lifestyles of Three *Trichoderma* Species. Microbiol. Mol. Biol. Rev..

[7] Park H.-S., Yu J.-H. (2012). Genetic control of asexual sporulation in filamentous fungi. Curr. Opin. Microbiol..

[8] Latgé J.-P., Beauvais A., Chamilos G. (2017). The Cell Wall of the Human Fungal Pathogen *Aspergillus fumigatus*: Biosynthesis, Organization, Immune Response, and Virulence. Annu. Rev. Microbiol..

[9] Gow N.A.R., Latge J.-P., Munro C.A. (2017). The Fungal Cell Wall: Structure, Biosynthesis, and Function. Microbiol. Spectr..

[10] Lee M.J., Sheppard D.C. (2016). Recent advances in the understanding of the *Aspergillus fumigatus* cell wall. J. Microbiol..

[11] D’Enfert C. (1997). Fungal Spore Germination: Insights from the Molecular Genetics of *Aspergillus nidulans* and *Neurospora crassa*. Fungal Genet. Biol..

[12] Momany M. (2002). Polarity in filamentous fungi: Establishment, maintenance and new axes. Curr. Opin. Microbiol..

[13] Sheppard D.C., Howell P.L. (2016). Biofilm Exopolysaccharides of Pathogenic Fungi: Lessons from Bacteria. J. Biol. Chem..

[14] Loussert C., Schmitt C., Prevost M.-C., Balloy V., Fadel E., Philippe B., Kauffmann-Lacroix C., Latgé J.P., Beauvais A. (2010). In vivo biofilm composition of *Aspergillus fumigatus*. Cell. Microbiol..

[15] Fontaine T., Beauvais A., Loussert C., Thevenard B., Fulgsang C.C., Ohno N., Clavaud C., Prevost M.-C., Latgé J.-P. (2010). Cell wall alpha1-3glucans induce the aggregation of germinating conidia of *Aspergillus fumigatus*. Fungal Genet. Biol..

[16] Miyazawa K., Umeyama T., Hoshino Y., Abe K., Miyazaki Y. (2022). Quantitative Monitoring of Mycelial Growth of *Aspergillus fumigatus* in Liquid Culture by Optical Density. Microbiol. Spectr..

[17] Müller H., Barthel L., Schmideder S., Schütze T., Meyer V., Briesen H. (2022). From spores to fungal pellets: A new high-throughput image analysis highlights the structural development of *Aspergillus niger*. Biotechnol. Bioeng..

[18] Latgé J.-P., Chamilos G. (2019). *Aspergillus fumigatus* and Aspergillosis in 2019. Clin. Microbiol. Rev..

[19] Bruder S., Reifenrath M., Thomik T., Boles E., Herzog K. (2016). Parallelised online biomass monitoring in shake flasks enables efficient strain and carbon source dependent growth characterisation of *Saccharomyces cerevisiae*. Microb. Cell Fact..

[20] Hartmann T., Dümig M., Jaber B.M., Szewczyk E., Olbermann P., Morschhäuser J., Krappmann S. (2010). Validation of a self-excising marker in the human pathogen *Aspergillus fumigatus* by employing the beta-rec/six site-specific recombination system. Appl. Environ. Microbiol..

[21] Schrettl M., Beckmann N., Varga J., Heinekamp T., Jacobsen I.D., Jöchl C., Moussa T.A., Wang S., Gsaller F., Blatzer M. (2010). HapX-mediated adaption to iron starvation is crucial for virulence of *Aspergillus fumigatus*. PLoS Pathog..

[22] Blatzer M., Barker B.M., Willger S.D., Beckmann N., Blosser S.J., Cornish E.J., Mazurie A., Grahl N., Haas H., Cramer R.A. (2011). SREBP Coordinates Iron and Ergosterol Homeostasis to Mediate Triazole Drug and Hypoxia Responses in the Human Fungal Pathogen *Aspergillus fumigatus*. PLoS Genet..

[23] Krappmann S., Sasse C., Braus G.H. (2006). Gene targeting in *Aspergillus fumigatus* by homologous recombination is facilitated in a nonhomologous end-joining-deficient genetic background. Eukaryot. Cell.

[24] Bertuzzi M., van Rhijn N., Krappmann S., Bowyer P., Bromley M.J., Bignell E.M. (2021). On the lineage of *Aspergillus fumigatus* isolates in common laboratory use. Med. Mycol..

[25] Nierman W.C., Pain A., Anderson M.J., Wortman J.R., Kim H.S., Arroyo J., Berriman M., Abe K., Archer D.B., Bermejo C. (2005). Genomic sequence of the pathogenic and allergenic filamentous fungus *Aspergillus fumigatus*. Nature.

[26] Pontecorvo G., Roper J.A., Hemmons L.M., MacDonald K.D., Bufton A.W.J. (1953). The genetics of *Aspergillus nidulans*. Adv. Genet..

[27] Bergmann A., Hartmann T., Cairns T., Bignell E.M., Krappmann S. (2009). A regulator of *Aspergillus fumigatus* extracellular proteolytic activity is dispensable for virulence. Infect. Immun..

[28] Cheung A.L., Ying P., Fischetti V.A. (1991). A method to detect proteinase activity using unprocessed X-ray films. Anal. Biochem..

[29] Schindelin J., Arganda-Carreras I., Frise E., Kaynig V., Longair M., Pietzsch T., Preibisch S., Rueden C., Saalfeld S., Schmid B. (2012). Fiji: An open-source platform for biological-image analysis. Nat. Methods.

[30] Quantification of Fluorescence Spots Intensity along The Spot Area. https://forum.image.sc/t/quantification-of-fluorescence-spots-intensity-along-the-spot-area/21522.

[31] R Core (2021). Team R: A Language and Environment for Statistical Computing.

[32] Wickham H., Averick M., Bryan J., Chang W., McGowan L.D., François R., Grolemund G., Hayes A., Henry L., Hester J. (2019). Welcome to the tidyverse. J. Open Source Softw..

[33] Wickham H. (2016). ggplot2: Elegant Graphics for Data Analysis.

[34] Kassambara A. (2020). ggpubr: ‘ggplot2’ Based Publication Ready Plots. https://CRAN.R-project.org/package=ggpubr.

[35] Schauberger P., Walker A. (2021). openxlsx: Read, Write and Edit xlsx Files. https://CRAN.R-project.org/package=openxlsx.

[36] Schrettl M., Bignell E., Kragl C., Sabiha Y., Loss O., Eisendle M., Wallner A., Arst H.N., Haynes K., Haas H. (2007). Distinct roles for intra- and extracellular siderophores during *Aspergillus fumigatus* infection. PLoS Pathog..

[37] Santamaria F., Reyes F. (1988). Proteases produced during autolysis of filamentous fungi. Trans. Br. Mycol. Soc..

[38] Latimer P., Pyle B.E. (1972). Light scattering at various angles. Theoretical predictions of the effects of particle volume changes. Biophys. J..

[39] Garcia-Rubio R., Monzon S., Alcazar-Fuoli L., Cuesta I., Mellado E. (2018). Genome-Wide Comparative Analysis of *Aspergillus fumigatus* Strains: The Reference Genome as a Matter of Concern. Genes.

[40] Keller N.P. (2017). Heterogeneity Confounds Establishment of “a” Model Microbial Strain. mBio.

[41] Sugui J.A., Pardo J., Chang Y.C., Müllbacher A., Zarember K.A., Galvez E.M., Brinster L., Zerfas P., Gallin J.I., Simon M.M. (2007). Role of *laeA* in the Regulation of *alb1*, *gliP*, Conidial Morphology, and Virulence in *Aspergillus fumigatus*. Eukaryot. Cell.

[42] Kowalski C.H., Beattie S.R., Fuller K.K., McGurk E.A., Tang Y.-W., Hohl T.M., Obar J.J., Cramer R.A. (2016). Heterogeneity among Isolates Reveals that Fitness in Low Oxygen Correlates with *Aspergillus fumigatus* Virulence. mBio.

